# Snakebites and resource utilization in pediatric urban and rural populations in the United States: 2016–2023

**DOI:** 10.1186/s40621-025-00563-3

**Published:** 2025-02-27

**Authors:** Kristyn Jeffries, Sara C. Sanders, Rachel Ekdahl, Dustin E. Williford, Maxwell Taylor, Charalene Fisher, Jacob Filipek, Brittany Slagle, Esma Birisci, Rebecca M. Cantu

**Affiliations:** 1 Department of Pediatrics, University of Arkansas for Medical Sciences, Little Rock, Arkansas, USA; 2 Arkansas Children’s Hospital, Little Rock, Arkansas, USA; 3 Faculty of Economics and Administrative Sciences, Department of Econometrics, Bursa Uludağ University, Bursa, Turkey

**Keywords:** Snakebites, Pediatric, Rural, Hospitalizations

## Abstract

**Background:**

Nearly 7,000 snakebite injuries are reported yearly in the United States, with almost one quarter of those in the pediatric population. Due to increased exposure to snakes, rural children may experience different clinical outcomes for snakebite injuries. The goal of this study was to examine differences in resource utilization of rural and urban pediatric patients with snakebite injuries.

**Methods:**

This is a retrospective cross-sectional study of patients aged 21 years and under presenting with venomous snakebites in the United States from January 1, 2016, through March 31, 2023, using the Pediatric Hospital Information System database and ICD-10 codes indicating snakebites. Comparisons were conducted to evaluate demographic and clinical characteristics in association with resource utilization and complications between patients living in rural areas and patients living in urban areas.

**Results:**

The study included 2,633 patients from 23 states. The median age was 9 years; 61% of patients were male. Most patients were in the South and over 70% resided in urban areas. 82% of the population was admitted to a hospital, with median length of stay 1.59 days. Compared to urban patients, rural patients were more likely to be admitted and receive antivenom but were less likely to have an intensive care unit admission and have abnormal coagulation studies.

**Conclusions:**

Rural pediatric patients with snakebites had different resource utilization and clinical complications than urban patients.

**Supplementary Information:**

The online version contains supplementary material available at 10.1186/s40621-025-00563-3.

## Background

In 2021, there were nearly 7,000 snakebites reported to the United States’ (U.S.) National Poison Data System (NPDS), with 1,651 occurring in children aged 19 or younger [1, 2, 3, 4]; however, these data are limited to the snakebite victims that seek medical care and are subsequently reported to Poison Control Centers and are likely an underestimation.

Snake species found in the U.S vary by geographic region, but most snakebites are attributed to the Crotalidae (pit viper) and Elapidae (coral snakes) families [5]. Crotalidae venom is hemotoxic, leading to consumptive coagulopathy or shock, while Elapid venom causes neurotoxicity [2]. While antivenom for both snake families are available in the U.S. and effective in significantly decreasing mortality for severe cases, indicators for use of the antivenom are poorly defined and the antivenom is costly to administer [5]. Additionally, more than 20% of pediatric patients who receive it are placed in observation status, which may lead to higher cost burden for these families [3].

Prior literature has shown that rural children in the U.S., who may have increased exposure to snakes, also experience significant health disparities for various outcomes [6, 7]. Few studies have focused on specific snakebite-related differences in this population, and rural children may disproportionately experience this financial burden associated with increased resource utilization [3]. The purpose of this study is to identify differences in resource utilization and clinical outcomes in rural and urban pediatric patients with snakebites in the US.

## Methods

### Study design

This is a retrospective cross-sectional study using data obtained from the Pediatric Hospital Information System (PHIS), an administrative database that contains inpatient, emergency department (ED), ambulatory surgery and observation encounter-level data from not-for-profit, tertiary care pediatric hospitals in the U.S. These hospitals are affiliated with the Children’s Hospital Association (Lenexa, KS). Data quality and reliability are assured through a joint effort between the Children’s Hospital Association and participating hospitals. For the purposes of external benchmarking, participating hospitals provide discharge/encounter data including demographics, diagnoses, and procedures. Nearly all of these hospitals also submit resource utilization data (e.g. pharmaceuticals, imaging, and laboratory) into PHIS. Data are de-identified at the time of data submission and are subjected to several reliability and validity checks before being included in the database [8]. For this study, data from 40 hospitals were included.

## Study population

We included patients 0–21 years of age hospitalized at a PHIS-participating hospital from January 1, 2016 to March 31, 2023, with a principal diagnosis of snakebite, using the International Classification of Diseases, Tenth Revision (ICD-10) codes indicating venomous snakebite (Supplemental Data). Patients through age 21 were included as the American Academy of Pediatrics, U.S. Department of Health, and the U.S. Food and Drug Administration define adolescence through age 21 [9].

Patient demographics included age, sex, race and ethnicity (a combined descriptor assigned by PHIS), and urban status (based on patient’s home ZIP code). Rurality was determined by rural-urban commuting area (RUCA) with the four-category classification based on ZIP code [10]; in this study, large rural, small rural, and isolated codes were grouped into a “rural” category and the urban codes are referred to as “urban”. Patients were excluded if rural status was unknown. Geographic region of the home ZIP code was grouped in the U.S. Census Bureau divisions: Northeast, Midwest, South and West [11]. Additional variables obtained were, intensive care unit (ICU) charge flag, mortality flag, length of stay (LOS) in days, visit type, admission date, presence of and reason for readmission within 30 days (both all cause readmission and same cause readmission with the same APR-DRG as index admission), and PHIS codes for antivenin/antivenom (antivenin unspecified, antivenin crotalidae polyvalent, and antivenin micrurus fulvius), antibiotics (identified by PHIS codes for anti-infective agents), presence of compartment syndrome (Supplemental Data) and abnormal coagulation studies (Supplemental Data).

## Study outcomes

The main outcomes were resource utilization including: hospital admission, ICU admission, LOS, antivenom receipt, antibiotic usage, and readmission. Secondary outcomes evaluated were clinical complications including: compartment syndrome, abnormal coagulation studies, and mortality.

### Statistical analysis

We summarized patient characteristics using descriptive statistics. Group comparisons between rural and urban children were performed using Chi square tests for categorical statistics and independent t-tests for continuous variables, as appropriate. We calculated odds ratio (OR) to compare the likelihood of clinical outcomes between rural and urban children. Statistical significance was set at *p* < 0.05. Statistical analysis was performed with GraphPad Prism version 10.1.2 for Windows (San Diego, California, USA).

## Results

The total study population was 2,633 with 1,591 (60.4%) males and a median age of 9 years (interquartile range [IQR] 5–13 years). Twenty-three states were represented in the study, with the majority of patients (2,224; 83.5%) in the South. The study population was predominantly non-Hispanic White (1,986 patients; 74.6%). Based on home ZIP code, 71.6% (1,908 patients) of the study population resided in urban areas. (Table 1). Overall, 81.8% (2,181) of patients were admitted to a hospital, with 62% of admitted patients being male. The average LOS of admitted patients was 1.59 days (95% confidence interval [CI] 1.52, 1.67). There were 240 readmissions within 30 days, accounting for 9% of the study population; of these, 57 (23.8% of readmissions, 2.1% of study population) were for the same APR-DRG as the index admission. One hundred ninety (79.2%) were readmitted after a hospital admission and 50 (20.8%) were readmitted after an ED visit. There was one death in the study population.

**Table 1:** Demographics of pediatric patients who presented to a PHIS hospital with a principal diagnosis of snakebite

	Total population	Rural	Urban
N	2633	725	1908
Age in years, median (IQR)	9 (5–13)	8 (5–12)	9 (5–13)
Age group <5y 5-12y 13-17y 18-21y	576 1371 665 20	176 389 155 5	400 982 510 15
Gender Male Female Unknown	1591 1041 1	453 272 0	1138 769 1
Race NH White NH Black Hispanic Asian Multiracial Other Unknown	1964 122 342 32 14 109 51	600 22 65 0 2 22 14	1364 99 277 32 12 87 37
Median [Mean] LOS (d)	1 [1.48]	1 [1.38]	1 [1.52]

*IQR = interquartile range; y = years; d = days; NH = Non-Hispanic. N* = 30 excluded for unknown ZIP/rural status

Rural patients were more likely to be admitted to the hospital than urban patients (90% vs. 78.8%, *p* < 0.0001, OR 2.43). Rural patients were less likely to incur ICU charges than urban patients (12% vs. 17%, *p* < 0.005, OR 0.67) and there was no difference in the median number of ICU days between rural and urban patients (4 days for both groups).

Antivenom was used more often in rural patients than urban (58.2% vs. 49.3%, *p* < 0.001, OR 1.43). There was no significant difference in antibiotic usage (rural 7.7% vs. urban 9/2%, *p* = 0.22).

Rural patients were significantly less likely to have abnormal coagulation studies than urban patients (5.5% vs. 9.3%, *p* < 0.01, OR 0.57). There was no significant difference in compartment syndrome rates between rural and urban patients (0.41% vs. 0.37%, *p* = 1).

There were no significant differences between rural and urban patients’ LOS, mortality rate, all-cause readmission, or same-diagnosis readmission. (Table 2).

**Table 2:** Differences in urban/sSuburban and rural patients with snakebites

	Rural (*n* = 725)	Urban (*n* = 1908)	*p*-value
Hospital Admission, *n (%)*	653 (90.1)	1503 (78.8)	**< 0.0001**
ICU Charges, *n (%)*	87 (12)	324 (17)	**0.002**
Antivenom Given, *n (%)*	422 (58.2)	940 (49.3)	**< 0.0001**
Antibiotics Given, *n (%)*	55 (7.6)	175 (9.2)	0.22
Readmission, *n (%)*	60 (8.3)	174 (9.1)	0.54
Abnormal Coagulation Labs, *n (%)*	40 (5.5)	178 (9.3)	**0.002**
Compartment Syndrome, *n (%)*	3 (0.4)	7 (0.37)	> 0.99
Mortality	0	1	> 0.99

*P* < 0.05 indicates significant difference and presented as bold within table

## Discussion

This retrospective database study examined the impact of rurality on the resource utilization of children with snakebites presenting to U.S. children’s hospitals over a seven-year period. Children with rural home ZIP codes were significantly more likely to be admitted to the hospital and receive antivenom but less likely to have ICU charges compared to children with urban ZIP code. There were no differences in readmission rates or mortality.

Many potential factors may contribute to these findings. As community hospital pediatric beds have decreased [7, 12], community providers may have less pediatric-specific experience and comfort and be more inclined to transfer pediatric snakebite patients to larger pediatric centers, potentially increasing travel-related costs and impacting other social determinants of health for rural families [7]. Our study population was from pediatric tertiary care centers and showed that patients with rural home ZIP codes were significantly more likely to be admitted to a hospital despite the majority of the study population having urban or suburban ZIP codes. While the study was not designed to assess more granular details of the history and medical decision-making, clinicians may have a lower threshold to admit or treat patients with longer transportation times to medical care in case of progression of symptoms or development of complications. Further, this could also potentially be due to the sickest of those presenting to non-trauma centers being transferred to tertiary care centers, as in our study, rural patients were more likely to receive antivenom.

Our study adds to the literature describing resource utilization after snakebite injuries [3, 13, 14, 15], while highlighting a particularly vulnerable population in rural areas. Due to limitations within our administrative database, we were not able to evaluate the underlying causes of this different resource utilization for rural patients, such as severity of bite, identification of snake species, and patient’s distance to health care. Subsequent analysis of these factors in the future could help guide specific interventions toward equitable outcomes. Further studies evaluating pediatric management algorithms in this rural population may also help standardize treatments and decrease associated costs for patients and families [2, 13].

This study utilizes a large population of children presenting to children’s hospitals in the U.S.; although children’s hospitals represent an estimated 30% of pediatric hospitalizations in 2012, complex injuries such as snakebites may be more likely to be referred to such centers [14]. Limitations of this study include an administrative database that includes mostly large free-standing children’s hospitals, more likely located in urban centers, which likely underestimates the total burden of snakebite injuries presenting to more regional, rural hospitals as prior studies have shown that most patients are able to discharge from the ED without transfer [4]. This study did not include transfer information, so antivenom and antibiotics administered prior to transfer to a study hospital could not be assessed. Additionally, patients seen in non-study hospitals with snakebites that did not require transfer to an included center are not represented in this study. This study focused exclusively on snake species and rural populations within the U.S. and may not be generalizable to other global populations, though has previously been discussed in literature [16, 17].

## Conclusions

Our findings may be helpful in directing education on management of pediatric snakebites as well as resource allocation such as antivenom to areas with higher volumes of pediatric snakebites. Future studies focused on rural pediatric patients with snakebites may provide more specific guidance to local and tertiary hospitals on optimal preparation for and management of these patients.

## Electronic supplementary material

Supplementary Material 1

## Data Availability

The datasets used and analyzed during the current study are available from the corresponding author on reasonable request.

## Abbreviations

U.S.: United States
NPDS: National Poison Data System
PHIS: Pediatric Hospital Information System
ED: Emergency department
ICD-10: International Classification of Diseases, Tenth Revision
RUCA: Rural-urban commuting area
ICU: Intensive care unit
LOS: Length of stay
OR: Odds ratio
IQR: Interquartile range
CI: Confidence interval
