# Supplementary material for: Snakebites and resource utilization in pediatric urban and rural populations in the United States: 2016–2023
Source: Inj Epidemiol. 2025 Feb 27;12:11. doi: 10.1186/s40621-025-00563-3 (PMC11866564; doi:10.1186/s40621-025-00563-3)
Supplement: Supplementary file 1 — Supplementary Material 1 [file 40621_2025_563_MOESM1_ESM.docx]

**Supplemental Data – ICD-10 codes**

| **ICD-10 CODE** | **DIAGNOSIS CODE TITLE** |
| --- | --- |
| **Snakebite** | |
| 9-E9050 | Venomous snake bite |
| 9-E9062 | Nonvenomous snake bite |
| T63.001A | Toxic effect of unspecified snake venom, accidental (unintentional), initial encounter |
| T63.002A | Toxic effect of unspecified snake venom, intentional self-harm, initial encounter |
| T63.003A | Toxic effect of unspecified snake venom, assault, initial encounter |
| T63.004A | Toxic effect of unspecified snake venom, undetermined, initial encounter |
| T63.011A | Toxic effect of rattlesnake venom, accidental (unintentional), initial encounter |
| T63.012A | Toxic effect of rattlesnake venom, intentional self-harm, initial encounter |
| T63.013A | Toxic effect of rattlesnake venom, assault, initial encounter |
| T63.014A | Toxic effect of rattlesnake venom, undetermined, initial encounter |
| T63.021A | Toxic effect of coral snake venom, accidental (unintentional), initial encounter |
| T63.022A | Toxic effect of coral snake venom, intentional self-harm, initial encounter |
| T63.023A | Toxic effect of coral snake venom, assault, initial encounter |
| T63.024A | Toxic effect of coral snake venom, undetermined, initial encounter |
| T63.031A | Toxic effect of taipan venom, accidental (unintentional), initial encounter |
| T63.032A | Toxic effect of taipan venom, intentional self-harm, initial encounter |
| T63.033A | Toxic effect of taipan venom, assault, initial encounter |
| T63.034A | Toxic effect of taipan venom, undetermined, initial encounter |
| T63.041A | Toxic effect of cobra venom, accidental (unintentional), initial encounter |
| T63.042A | Toxic effect of cobra venom, intentional self-harm, initial encounter |
| T63.043A | Toxic effect of cobra venom, assault, initial encounter |
| T63.044A | Toxic effect of cobra venom, undetermined, initial encounter |
| T63.061A | Toxic effect of venom of other North and South American snake, accidental (unintentional), initial encounter |
| T63.062A | Toxic effect of venom of other North and South American snake, intentional self-harm, initial encounter |
| T63.063A | Toxic effect of venom of other North and South American snake, assault, initial encounter |
| T63.064A | Toxic effect of venom of other North and South American snake, undetermined, initial encounter |
| T63.071A | Toxic effect of venom of other Australian snake, accidental (unintentional), initial encounter |
| T63.072A | Toxic effect of venom of other Australian snake, intentional self-harm, initial encounter |
| T63.073A | Toxic effect of venom of other Australian snake, assault, initial encounter |
| T63.074A | Toxic effect of venom of other Australian snake, undetermined, initial encounter |
| T63.081A | Toxic effect of venom of other African and Asian snake, accidental (unintentional), initial encounter |
| T63.082A | Toxic effect of venom of other African and Asian snake, intentional self-harm, initial encounter |
| T63.083A | Toxic effect of venom of other African and Asian snake, assault, initial encounter |
| T63.084A | Toxic effect of venom of other African and Asian snake, undetermined, initial encounter) |
| T63.091A | Toxic effect of venom of other snake, accidental (unintentional), initial encounter |
| T63.092A | Toxic effect of venom of other snake, intentional self-harm, initial encounter |
| T63.093A | Toxic effect of venom of other snake, assault, initial encounter |
| T63.094A | Toxic effect of venom of other snake, undetermined, initial encounter |
| W59.11XA | Bitten by nonvenomous snake, initial encounter |
| W59.12XA | Struck by nonvenomous snake, initial encounter |
| W59.19XA | Other contact with nonvenomous snake, initial encounter |
| E-9050 | Venomous snakes and lizards causing poisoning and toxic reactions |
| **Compartment Syndrome** | |
| T79A0XA | Compartment syndrome, unspecified, initial encounter |
| T79A11A | Traumatic compartment syndrome of right upper extremity, initial encounter |
| T79A12A | Traumatic compartment syndrome of left upper extremity, initial encounter |
| T79A19A | Traumatic compartment syndrome of unspecified upper extremity, initial encounter |
| T79A21A | Traumatic compartment syndrome of right lower extremity, initial encounter |
| T79A22A | Traumatic compartment syndrome of left lower extremity, initial encounter |
| T79A29A | Traumatic compartment syndrome of unspecified lower extremity, initial encounter |
| T79A3XA | Traumatic compartment syndrome of abdomen, initial encounter |
| T79A9XA | Traumatic compartment syndrome of other sites, initial encounter |
| 729.71 | Nontraumatic compartment syndrome of upper extremity |
| 729.72 | Nontraumatic compartment syndrome of lower extremity |
| 729.73 | Nontraumatic compartment syndrome of abdomen |
| 729.79 | Nontraumatic compartment syndrome of other sites |
| 958.90 | Compartment syndrome, unspecified |
| 958.91 | Traumatic compartment syndrome of upper extremity |
| 958.92 | Traumatic compartment syndrome of lower extremity |
| 958.93 | Traumatic compartment syndrome of abdomen |
| 958.99 | Traumatic compartment syndrome of other sites |
| **Abnormal Coagulation Studies** | |
| 790.92 | Abnormal coagulation profile |
| R.791 | Abnormal coagulation profile, not otherwise specified |
| 286.9 | Other and unspecified coagulation defects |
| D65 | Disseminated intravascular coagulopathy |
| D68.9 | Coagulation defect, unspecified |
| 287.4 | Secondary thrombocytopenia |
| 287.49 | Other secondary thrombocytopenia |
| 287.5 | Thrombocytopenia, unspecified |
| D69.59 | Other secondary thrombocytopenia |
| D69.6 | Thrombocytopenia, unspecified |
